# Autologous microfragmented adipose tissue treatment of knee osteoarthritis demonstrates effectiveness in 68% of patients at 4-year follow-up

**DOI:** 10.1007/s00402-023-05143-y

**Published:** 2024-01-11

**Authors:** Francesco Onorato, Massimiliano Rucci, Mattia Alessio-Mazzola, Alessandro Bistolfi, Carlotta Castagnoli, Matteo Formica, Riccardo Ferracini

**Affiliations:** 1 Department of Orthopedics and Traumatology, Orthopedic and Trauma Center, Città della Salute e della Scienza di Torino, University of Turin, Via Zuretti 29, 10126 Turin, Italy; 2 Department of Surgical Sciences (DISC), Orthopaedics and Traumatology Clinic, Ospedale Policlinico San Martino, University of Genoa, Largo Rosanna Benzi 10, 16132 Genova, Italy; 3 IRCCS Ospedale San Raffaele, Orthopaedic and Trauma Unit, Via Olgettina 60, 20132 Milan, Italy; 4 Orthopaedics and Traumatology, Ospedale Cardinal Massaia Asti, Via Conte Verde 125, 14100 Asti, Italy; 5 Department of General Surgery and Special Surgery, Burns Center Unit, Unit of Skin Bank, Via Zuretti 29, 10126 Turin, Italy; 6 Department of Orthopedics and Traumatology, Ospedale Koelliker, Corso Galileo Ferraris 247/255, 10134 Turin, Italy

**Keywords:** Knee osteoarthritis, Adipose tissue-derived mesenchymal stem cells, Intra-articular injection, Medium term follow-up

## Abstract

**Background:**

Adipose tissue-derived stem cells are an interesting therapeutic option for early knee osteoarthritis (OA) treatment due to their high plasticity, easiness of harvesting and rapidity of administration. The aim of this study was to evaluate the medium-term effectiveness and safety of Microfragmented Autologous Fat Tissue (MFAT) injection treatment at 4-year follow-up and to investigate potential correlations among patients’ pre-treatment clinical condition and clinical outcomes to identify possible predicting factors for procedure success or failure.

**Patients and methods:**

This is a prospective trial enrolling 46 patients with diagnosis of symptomatic knee OA and failure of previous conservative measures who underwent diagnostic arthroscopy and single autologous MFAT injection between June 2017 and July 2018. Patients were assessed with repeated clinical scoring systems at baseline, 6 months, 1 and 4 years after surgery. The evaluation included demographic characteristics, arthroscopic findings, and stem cell number from injected tissue.

**Results:**

No major complications were reported during follow-up period and there was a significant increase of Lysholm knee score from baseline value of 61.7 ± 13.8 to 79.5 ± 16.9 at 4 years (*p* < 0.001). The WOMAC score increased from a baseline value of 66.5 ± 14.7 to 82.8 ± 15.7 at 4 years (*p* < 0.001) and there was a significant decrease of VAS pain score from baseline value of 6.3 ± 1.5 to 3.5 ± 2.6 at 4-year follow-up (*p* < 0.001). ROM improved significantly from 118.4 ± 2.6 to 122.5 ± 2.5 at 12 months (*p* < 0.001), but did not improve at 4 years (*p* > 0.05). 15 patients (32.6%) were considered treatment failures, because they required secondary surgery, further injection therapy or experienced symptoms persistence. Patient with synovitis had 75% failure rate, although synovitis did not result as a statistically significant factor influencing clinical outcome up to 4-year follow-up (*p* = 0.058). Age, cartilage defects severity, BMI, concomitant procedures, and stem cell number from injected MFAT did not show any significant correlation with the results.

**Conclusions:**

MFAT intra-articular injection is a safe procedure with positive improvements up to 4-year follow-up in patients with early knee OA. These findings suggest MFAT could be a minimally invasive treatment of early knee OA with durable benefits at mid-term evaluation.

**Trial registration:**

IRB number ID-3522.

## Background

Knee Osteoarthritis (OA) is one of the most common chronic diseases worldwide leading to pain and functional disability. Prevalence is expected to progressively increase due to aging of the population and the persistent increase of obesity and sedentary lifestyle [1–3]. Whenever failure of symptomatic medical therapy occurs, there is a lack of durable effective alternative treatment before joint irreversible degeneration, when total knee replacement (TKR) remains the only surgical option. Moreover, many knee OA patients are either too young or have a high functional demand to undergo joint replacement surgery [4].

Since traditional intra-articular injectable therapies demonstrated poor and short-lasting clinical improvements, biologic therapies ranging from platelet-rich plasma (PRP) to cell-based treatments have been introduced in clinical practice due to their attractive potential of inducing tissue healing and anti-inflammatory effects, without being invasive [5–8]. Among the biologic agents, mesenchymal stem cells (MSCs) extracted from different tissues have shown homeostatic effects on cartilage and joint. MSCs showed high plasticity, self-renewal capacity, multi-lineage differentiation potential as well as immuno-modulatory, metabolic, and bioactive actions through secretion of different growth factors and cytokines [9–13]. Since the pathophysiology of OA is based on both degenerative and inflammatory components, MSCs have been evaluated as a possibly disease-modifying treatment [14]. Adipose tissue-derived stem cells (ADSCs) represent an attractive source of MSCs due to easiness of harvesting and handling; in addition, they are not affected by aging and showed chondrogenic differentiation both in vitro and in vivo under adequate conditions [15]. Nevertheless, clinical use of ADSCs is strictly regulated by Food and Drugs Administration (FDA) and European Medical Agency (EMA), causing these products to be restricted when subjected to enzymatic tissue digestions [16–18]. This regulatory burden has led to novel therapeutic options considering “minimal manipulation” within or “nearby” the operating room as an approach to adhere to the regulations [15, 19]. The development of new medical systems allows to minimally manipulate adipose tissue, mostly through mechanical fragmentation or filtration preserving its structural niches. The biologic extract is then reinjected in the joint, exhibiting effectiveness [20, 21]. According to our previous research, ADSCs express markers such as CD105 + , CD73 + , CD90 + and CD44 + and negative for HLA-DR, CD34, CD31 and CD45 [22].

Several randomized controlled trials (RCTs) have demonstrated the safety and efficacy of intra-articular injection of ADSCs with promising results; however, these studies were mostly on short-term follow-up [23–25].

The aim of the present study was to evaluate the effectiveness and safety of a single dose of Microfragmented Autologous Fat Tissue (MFAT) injection treatment in a cohort of patients affected by mild to moderate knee OA at 4-year follow-up. The second purpose was to investigate potential correlations among patients’ pre-treatment clinical conditions and clinical outcomes to identify possible predicting factors for success or failure of the procedure at mid-term evaluation.

## Materials and methods

### Study design and patient selection

We designed a prospective trial including 50 consecutive patients with knee osteoarthritis and failure of all previous conservative measures (physiotherapy, anti-inflammatory drugs, analgesics, hyaluronic acid, corticosteroid and PRP injection). Ethical approval for the present study was requested and obtained from the institutional review board (IRB number ID-3522).

Patients underwent diagnostic arthroscopy and MFAT injection treatment between June 2017 and July 2018. All cases were treated by a single operator at two different institutions. Before being included in the study, all patients gave their written informed consent which followed the Strengthening the Reporting of Observational Studies in Epidemiology (STROBE) guidelines and checklist.

Study population was selected according to the following inclusion criteria: knee pain and functional impairment, evidence of mild to moderate knee OA stages II–III according to Kellgren–Lawrence (K–L) either of the medial or lateral compartment at standard X-ray examination, presence of knee cartilage defects at 1.5 T magnetic resonance imaging (MRI) assessment, and failure of all conservative measures in the previous 18 months [26]. Patients were excluded in case of pregnancy or lactation, history of rheumatic diseases, coagulative disorders, history of alcohol or substance abuse, knee joint infections, skin disease or infections within the area of the injection site, diabetes, previous articular knee fractures, lower limb malalignment > 8°, MRI evidence of concurrent anterior cruciate ligament tear as well as clear evidence of meniscal tear. Patients with severe medical illness with American Society of Anaesthesiologists (ASA) score > 3 were also excluded [27].

All patients were clinically evaluated before the injective procedure and at the follow-up visits at 6, 12 months and 4 years after surgery. Arthroscopic evidence of chondral damage was described through International Cartilage Repair Society (ICRS) scoring grade [28]. The clinical outcome was assessed through Western Ontario and McMaster Universities Arthritis Index Score (WOMAC) [29], Lysholm Knee Score [30] and range of motion (ROM) to assess knee functional disability. The visual analogue scale (VAS) [31] was used to assess subjective level of pain. Primary outcome of the study was defined as a significant difference between baseline reported scoring values and corresponding 4-year results. Secondary outcome was the assessment of the influence of patients demographic features on the clinical results.

Among study group, a preliminary double-blind imaging evaluation was performed by two independent radiologists comparing pre-treatment and 4-year follow-up X-rays.

Failure was defined as secondary knee replacement surgery and further injection therapy, because symptoms recurrence or clinical scores not reaching Minimal Clinically Important Improvement (MCII) defined in this study as 5 points difference in WOMAC score during the follow-up visits [32]. The scores of the failed patients were included in the statistical assessment of outcome scores until the failure endpoints.

Treatment safety, complications and adverse events were assessed during follow-up visits. Serious adverse events were defined as conditions requiring hospitalization or intervention, while minor adverse events were defined as persistent knee pain or swelling and bruise or lipoatrophy at harvesting site.

### Microfragmented autologous fat tissue preparation and diagnostic arthroscopy

The procedure was performed with the patient in supine position under spinal anaesthesia. Harvesting of adipose tissue was performed under sterile conditions within the operating theatre. Lower abdomen was the favourite donor site but in case of low-fat mass, harvesting was performed from the lateral thigh subcutaneous tissues. The anatomical landmark for the small incision in the standard technique was 2 or 3 cm proximal to the pubic symphysis on the umbilical line. The adipose tissue was harvested through a blunt cannula after the injection of approximately 300 mL of Ringer solution containing 0.5 mg of adrenaline to reduce bleeding (modified Klein solution) [33]. Ten minutes after the infusion, approximately 100 mL of fat tissue were harvested and connected to a Vaclock® 20 ml syringe. At the end of the procedure, the skin was closed with steri-strips. All patients were invited to wear an abdominal compression bandage for 3 weeks.

Preparation of MFAT tissue aspirate was performed using the Lipogems® kit system that mechanically fragments adipose tissue clusters through constant irrigation with saline solution allowing removal of blood residues and tissue debris. The obtained micro-fragmented tissue was collected in sterile 10 ml syringes for injection in the affected knee. MFAT injection was performed at the end of diagnostic knee arthroscopy through one of the two arthroscopic portals under direct visualization. During the associated knee arthroscopic procedure, diagnostic assessment of intra-articular disorders including meniscal, articular cartilage lesions and evidence of synovitis characterized by an increased vascularity was executed and meniscus shaving performed when arthroscopic evidence of degeneration was accidentally found [34]. Between 60 and 20 mL of concentrated lipoaspirate volumes for each patient were delivered to Tissue and Cell Factory Skin Bank of Turin for cell analysis. The percentage of ADSCs in the MFAT volume was determined, and then the number of ADSCs for ml of MFAT was normalized on the absolute number of viable cells.

### Statistical analysis

All statistical analyses were performed using GraphPad Prism 9.0 (San Diego, California, USA).

A power analysis was conducted to calculate the sample size. According to the literature [35], with the Lysholm value of 56.2 ± 12.1 points for patients affected by mild to moderate knee osteoarthritis for a statistical power of 80% and a level of significance of 0.05, a total of 38 cases are needed to observe a significant difference between the baseline and the final values of Lysholm score. Considering a possible dropout of 25%, 50 patients were prospectively enrolled. The Shapiro–Wilk test was used to calculate normal distribution of variables. Categorical variables are expressed as absolute number of cases and relative percentage. Continuous variables are expressed with means and standard deviation with range and 95% confidence intervals (95% CI). Continuous variables of independent groups were compared with Mann–Whitney *U* test. Categorical variables were compared using the Chi-square test. The repeated measures analysis of variance (ANOVA) with Post Hoc test was used to compare outcome values at different timepoints and to assess correlation between continuous variables. Survival data were investigated with a log-rank test for equality of survivorship with Kaplan–Meier curves generated using subsequent surgery and poor clinical outcome as endpoint. For all variables, statistical significance was set at *p* < 0.05.

## Results

### Study population

From June 2017 to July 2018, 50 patients were prospectively enrolled and underwent MFAT procedure, 4 patients were lost to follow-up; therefore, 46 subjects were finally included in the study. Table 1 reports the demographic features of the study population. Four patients (8.7%) had history of previous surgery on the treated knee (3 medial and 1 lateral arthroscopic partial meniscectomies). All 46 patients underwent MFAT injection combined with diagnostic arthroscopy of whom 14 (30.4%) with concurrent meniscal shaving due to accidental arthroscopic degenerative findings (12 medial and 2 lateral). Arthroscopic diagnostic data of the studied population are summarized in Table 2. All patients received a single knee intra-articular injection of MFAT.

**Table 1:** Study population

Gender (female/male)	27 (58.7%)/19 (41.3%)
Side (left/right)	23 (50.0%)/23 (50.0%)
Age at surgery (years)	59.1 ± 9.8 (range: 31–75; 95% CI 56.3–61.9)
ADSC number	2.64 × 10^6^ ± 5.5 × 10^6^ (range: 2.6 × 10^3^ to 2.45 × 10^7^; 95% CI 1.03 × 10^6^–4,24 × 10^6^)
BMI	25.3 ± 3.5 (range: 20–37.1; 95% CI 24.3–26.3)
Previous surgery on index knee	4 (8.7%) (3 AMM, 1 ALM)

*AMM* arthroscopic medial meniscectomies, *ALM* arthroscopic lateral meniscectomies, *ADSC* adipose-derived stem cells, *MFAT* microfragmented adipose tissue

**Table 2:** Arthroscopic findings

Cartilage ICRS grade II	15 (32.6%)
Cartilage ICRS grade III	23 (54.8%)
Cartilage ICRS grade IV	8 (16.6%)
Cartilage damage location	
Medial compartment	18 (39.1%)
Isolated patello-femoral	9 (19.6%)
Medial + patello-femoral	8 (17.4%)
Medial + lateral	4 (9.5%)
Lateral	3 (7.1%)
Lateral + patello-femoral	3 (7.1%)
Diffuse	1 (2.1%)
Synovitis + cartilage damage	4 (9.5%)
Absence of synovitis	42 (91.3%)

*ICRS* International Cartilage Repair Society

### Clinical results

**Fig. 1:**
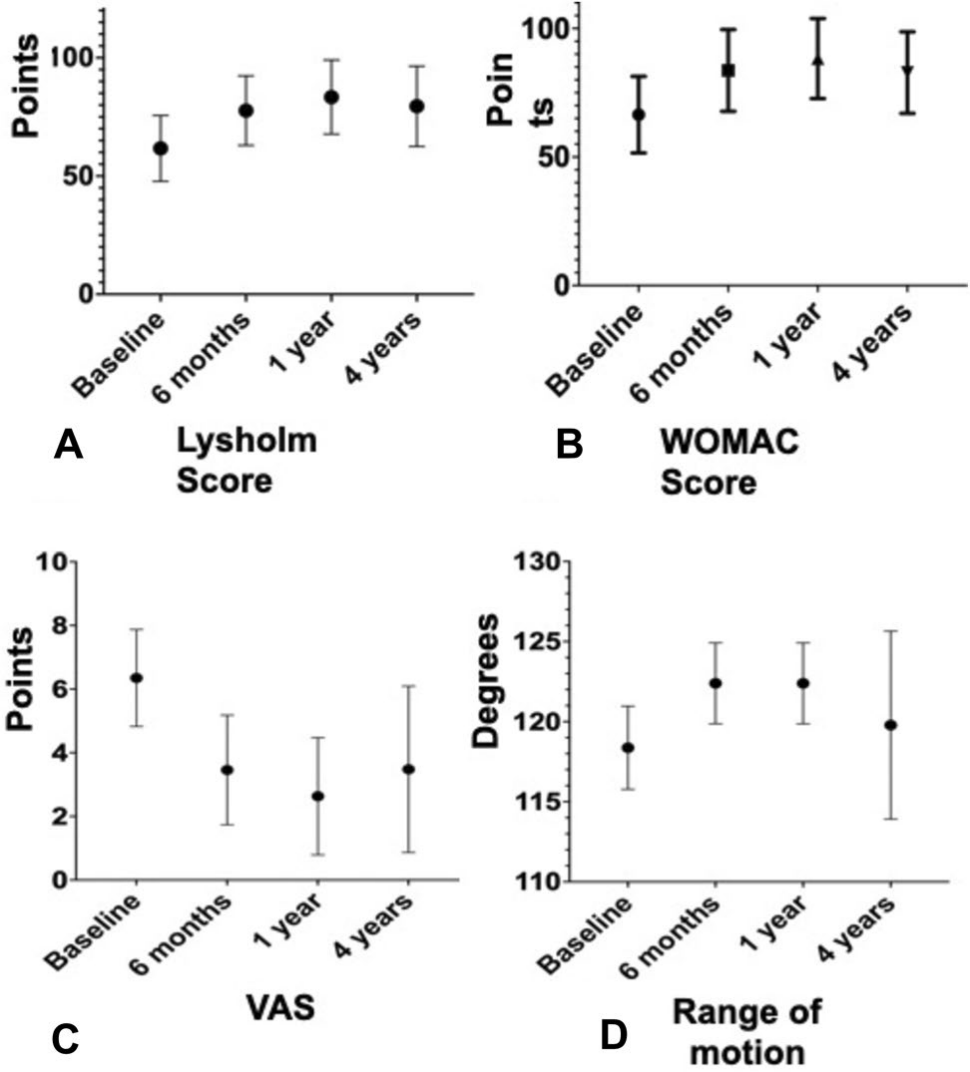
Patients reported outcome measures and range of motion (ROM) at baseline and at 6, 12 months and 4 years of follow-up

As previously hypothesized in the “Materials and Methods” section, an overall significant improvement of included outcome measures was observed at all clinical assessments. There was a significant improvement of Lysholm knee score from baseline value of 61.7 ± 13.8 to 79.5 ± 16.9 at 4-year follow-up (*p* < 0.001). WOMAC score improved significantly from baseline value of 66.5 ± 14.7 to 82.8 ± 15.7 at 4 years (*p* < 0.001). ROM increased significantly from 118.4 ± 2.6 to 122.5 ± 2.5 at 12-month follow-up (p < 0.001) but did not improve at 4-year follow-up (*p* = 0.305). There was a significant decrease in VAS for pain from baseline value of 6.3 ± 1.5 to 3.5 ± 2.6 at 4-year follow-up (*p* < 0.001). WOMAC (*p* = 0.021) was the only clinical outcome score showing a significant decline between 12 months and 4-year assessments. In addition, ROM (*p* = 0.002) showed a significant decline from 6 and 12 months and 4-year follow-up. Details of measured outcome values are reported in Tables 3, 4, 5and6 and Fig. 1.

**Table 3:** Prospective comparisons of Lysholm outcome values at different timepoints of follow-up

Lysholm score	Baseline	6 months	12 months	48 moths
Mean (SD)	61.7 (13.8)	77.7 (14.5)	83.4 (15.5)	79.5 (16.9)
Range	40.0–99.0	37.2–95.0	40.0–100.0	41.0–100.0
95% CI	57.7–65.7	73.5–81.9	78.9–87.9	74.6–84.3

		Mean difference	SE	*p*
Baseline	6 months	− 15.98	2.6	< 0.0001*
Baseline	12 months	− 21.72	2.7	< 0.0001*
Baseline	48 months	− 17.82	3.2	< 0.0001*
6 months	12 months	− 5.73	0.2	< 0.0001*
6 months	48 months	− 1.84	1.9	0.779
12 months	48 months	3.89	2.0	0.2158

*SD* standard deviation, *CI* confidence interval, *SE* standard error of difference

**Table 4:** Prospective comparisons of WOMAC outcome values at different timepoints of follow-up

Womac score	Baseline	6 months	12 months	48 months
Mean (SD)	66.5 (14.7)	83.7 (15.7)	88.3 (15.4)	82.8 (15.7)
Range	43.5–100.0	40.4–100.0	43.5–100	50.8–100.0
95% CI	62.3–70.8	79.2–88.2	83.9–92.8	78.3–87.3

Womac score		Mean difference	SE	*P*
Baseline	6 months	− 17.2	2.8	< 0.0001 *
Baseline	12 months	− 21.9	2.7	< 0.0001 *
Baseline	48 months	− 16.3	3.0	< 0.0001 *
6 months	12 months	− 4.6	0.4	< 0.0001 *
6 months	48 months	0.9	1.8	0.964
12 months	48 months	5.5	1.8	0.021 *

*SD* standard deviation, *CI* confidence interval, *SE* standard error of difference

**Table 5:** Prospective comparisons of VAS outcome values at different timepoints of follow-up

VAS	Baseline	6 months	12 months	48 months
Mean (SD)	6.3 (1.5)	3.5 (1.7)	2.6 (1.8)	3.5 (2.6)
Range	1.0–8.0	1.0–8.0	1.0–8.0	0.0–9.0
95% CI	5.8–6.7	3.0–3.9	2.1–3.1	2.7–4.3

VAS score		Mean difference	SE	*P*
Baseline	6 months	2.9	0.3	< 0.0001 *
Baseline	12 months	3.7	0.3	< 0.0001 *
Baseline	48 months	2.9	0.5	< 0.0001 *
6 months	12 months	0.8	0.1	< 0.0001 *
6 months	48 months	− 0.02	0.4	0.999
12 months	48 months	− 0.9	0.3	0.08

*SD* standard deviation, *CI* confidence interval, *SE* standard error of difference

**Table 6:** Prospective comparisons of ROM values at different timepoints of follow-up

ROM	Baseline	6 months	12 months	48 months
Mean (SD)	118.4 (2.6)	122.4 (2.5)	122.5 (2.5)	119.8 (5.8)
Range	105.0–125.0	120.0–125.0	120.0–125.0	95.0–125
95% CI	117.6–119.5	121.7–123.1	121.7–123.2	118.1–121.4

ROM		Mean Difference	SE	*P*
Baseline	6 months	− 4.0	0.3	< 0.0001*
Baseline	12 months	− 4.0	0.3	< 0.0001*
Baseline	48 months	− 1.4	0.8	0.3054
6 months	12 months	0.0	0.0	1.000
6 months	48 months	2.6	0.8	< 0.002*
12 months	48 months	2.6	0.8	< 0.002*

Fifteen patients (32.6%) demonstrated failure criteria at 4-year follow-up: 7 patients (15.2%) had subsequent surgery because of worsening reported condition, 4 patients (8.7%) underwent further injection therapy due to symptoms recurrence, and other 4 patients (8.7%) did not reach MCII at final follow-up evaluation. Patients who failed did not exhibit significant differences among males and females (*p* = 0.607), BMI > 25 (*p* = 0.913) and age > 65 (*p* = 0.753) for all assessed clinical outcomes.

**Fig. 2:**
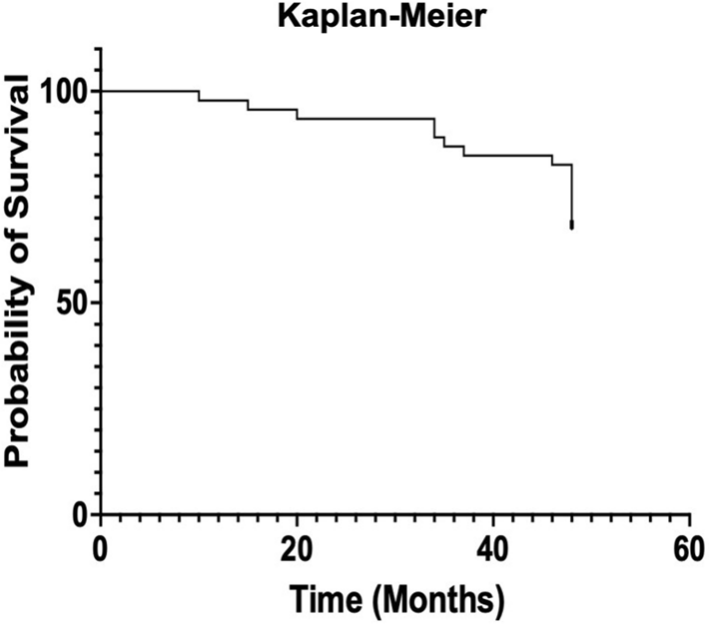
Kaplan–Meier survivorship function showing failure trend in study population

Kaplan–Meier survival function demonstrates the cumulative failure incidence and the trends up to 4 years (Fig. 2).

**Fig. 3:**
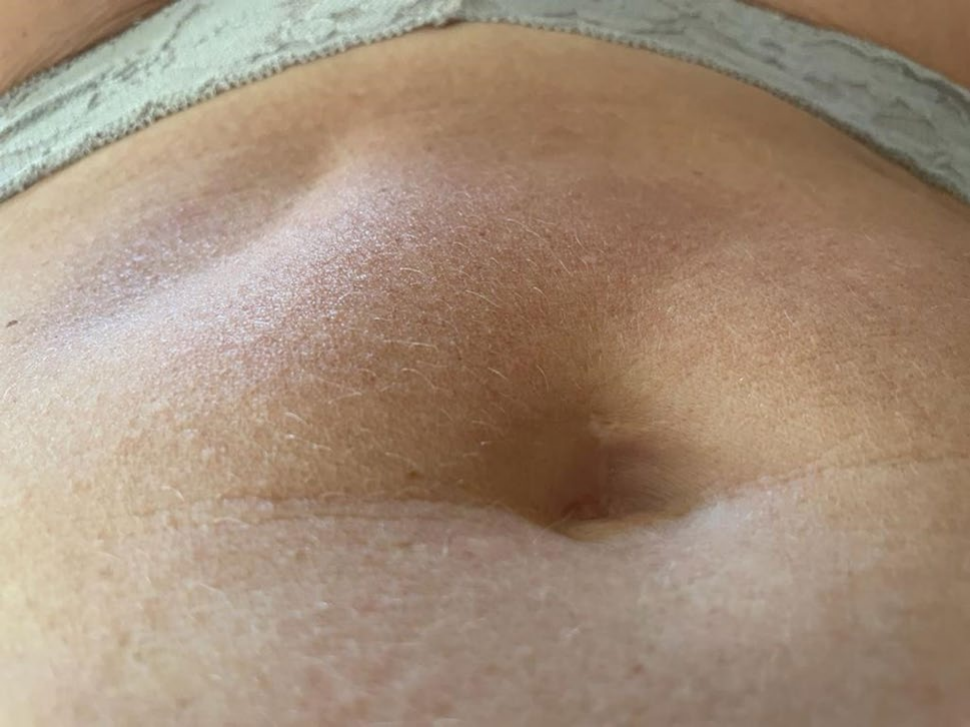
Lipoatrophy at the harvesting site 4 years after treatment

No major complications and adverse events were reported during follow-up evaluation. Five patients (10.9%) experienced persistent lipoatrophy at harvest site, but without significant difference in measured clinical outcome at any timepoints (Fig. 3).

Analysis performed to determine the parameters that influenced the clinical outcomes demonstrate that gender, BMI, age, previous knee surgery and ADSCs dose did not significantly correlate with outcome values at all timepoints (*p* > 0.05). Patients with arthroscopic evidence of synovitis did not demonstrate significantly lower outcome scores at 6 months, 1 year and at 4-year endpoint for all clinical scores (*p* > 0.05), even though they showed an “almost significant” difference in 4-year failure rate 75% (*p* = 0.058).

In this study group, there were no differences in outcome at all timepoints between patients with cartilage defects in different locations. The only exception was Lysholm score which differed significantly between patients with lateral defect and patients with medial plus patellofemoral defects (*p* = 0.034) at 6 months and the single patient with diffuse cartilage defect at 4 years (*p* = 0.0004). Chondral damage grade did not significantly affect clinical assessed outcomes at any timepoints among patients who had isolated or combined specific cartilage damage (*p* > 0.05). Similarly, concomitant arthroscopic meniscal shaving (medial or lateral) did not significantly influence the clinical outcome values at all timepoints (*p* > 0.05).

**Fig. 4:**
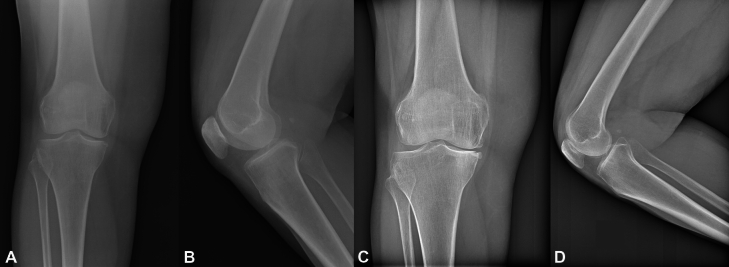
X-ray evaluation at baseline and at 4 years of follow-up after MFAT treatment in a 61-year-old woman with predominant medial knee OA. Minimal joint space narrowing, small osteophytes formation and subchondral sclerosis was found in the absence of clinical evolution at 4-year follow-up

Two double-blind trained radiologists evaluated X-ray imaging without reporting any relevant differences in K–L stage with respect to pre-operative X-ray in patients responding to treatment at 4 years (Fig. 4).

## Discussion

The main finding of the present study was a clinically meaningful improvement in knee OA symptoms from pre-treatment condition to 4-year follow-up after intra-articular injection of MFAT extracts. However, mid-term outcome scores at 4 years demonstrated to significantly decline as compared to those at 1 year in WOMAC score and in ROM.

To our knowledge, only one clinical trial in literature reported safety and potential efficacy of ADSCs infiltration over 4-year follow-up, most of them in fact monitored patients over a maximum period of a couple of years. Kim et al. documented a significant improvement in VAS and WOMAC scores of eleven patients up to 5-year follow-up, with imaging improvement in whole-organ magnetic resonance imaging scores (WORMS) up to 3 years after the injection [36, 37].

ADSCs are considered a promising product among the orthobiologics currently available, being easy to obtain with a minimally invasive procedure. In addition, according to Mazini et al. ADSCs have an even greater regenerative profile than bone-marrow-derived MSCs [38]. Literature already reported several clinical trials and metanalyses demonstrating substantial improvements of clinical symptoms and paucity of adverse events in knee OA patients infiltrated with ADSCs [39–41]. Although clinical results look positive, when assessing the technical procedures, it becomes apparent that high heterogeneity in the preparation method and dose of administered cells, as well as the frequent concomitant surgical procedure prevent us from a clear understanding of the real clinical potential of these stem cell-based products [19, 42, 43].

In this study three subjective knee scores (WOMAC, Lysholm and VAS) changed in time with similar trends: a progressive significant improvement in function, pain, and activity level up to 1 year with a subsequent small reduction up to 4 years. However, clinical scores firmly remained significantly improved as compared to those before the procedure with an overall acceptable satisfaction rate at final follow-up. Similarly, Kim et al. described declining trends of results after 3-year follow-up [36]. In this series second-look arthroscopy to assess intra-articular environment was not performed. However, Koh et al. in their second-look arthroscopy described an improved or stable cartilage arthroscopic status for at least 2 years postoperatively after ADSCs infiltration in patients over 65 affected by moderate knee OA [44].

In this clinical trial, adipose tissue infusion was not associated with any adverse event, such as chondro-toxicity, allergies, or implant rejection in the post-surgical period, confirming short-term data already reported in the literature [41]. Surprisingly, despite a quite heterogeneous population we did not find any influence of grade of cartilage defects on the outcome scores. We believe that the small population in the study did not allow us to reach enough statistical power, as half our patients (4/8) with cartilage defects grade IV ICRS failed. Furthermore, a recent work showed that patients with stage II K–L knee OA were most likely to benefit from intra-articular injection of ADSCs compared to the patients with stage IV K–L [45]. Similarly, Migliorini et al. showed that patients treated at earlier-degeneration stages reported statistically significant improvements of VAS, WOMAC and walking distance [46]. Other variables such as gender, BMI, sex and previous surgeries had no influence on clinical outcomes values, reinforcing the hypothesis of MFAT effectiveness. Despite the possible misleading response, we performed associated arthroscopic procedures to well-document cartilage defects size and grade (not clearly definable on MRI), to infer possible statistical correlations and to remove other potential pro-inflammatory sources of pain which could limit the immune-regulatory effect of the treatment [47]. Vasso et al. similarly documented an improvement in clinical functional scores in patients affected by patellofemoral OA without any influence by age, BMI, or stage of OA [48]. Our differences in Lysholm score regarding the site of chondral lesion are isolated findings and, therefore, could not lead to any conclusion. In the present trial ADSCs dose was not correlated with clinical outcomes, regardless of the wide range of stem cells quantity administered. In contrast, a recent prospective study described that clinical outcome of medium and low-dose ADSCs injection cohort deteriorated after 1 year, whereas in those patients within the high-dose group were preserved until 2 years [49]. However, most recent meta-analysis regarding clinical trials for the treatment of knee OA with autologous MSCs, did not document a clear dose response effect [41, 43].

An interesting aspect observed in literature, regards the impact of arthroscopic evidence of synovial inflammation which seems to prevent recovery function. In this series, no significant correlation was found between synovitis and outcome scores, even though among the four patients displaying arthroscopic evidence of synovial inflammation, three of them ended up with joint replacement at 4-year final assessment (75%). Therefore, the “almost significant” results regarding failure rate of patients with evidence of synovitis could be just related to the small dimension of the study cohort. Low grade synovial inflammation is a key aspect of the evolution of OA and might negatively affect anti-inflammatory function of ADSCs, therefore, could hinder therapeutic effect, as supported by in-vitro studies [22, 50]. Our hypothesis is that synovitis might hinder the mesenchymal and immunocompetent fat-derived cells from adhering and growing within their niches, therefore, preventing their biological functions. Nevertheless, further clinical trials might confirm this hypothesis.

Study treatment showed a failure rate of 15/46 patients (32.6%) at 4 years. Kim et al. reported different results; however, they considered improvements also patients undergoing additional intra-articular hyaluronic acid injection which we considered instead evidence of treatment failure [36]. Several patients had mild knee swelling associated with pain during the first week after surgery which then spontaneously resolved with analgesics. Five patients (10.9%) of our cohort reported persistent lipodystrophy without any functional alteration. To our knowledge, this is the first documentation of lipoatrophy at the harvesting site in literature [21]. This finding might be considered when illustrating possible long-lasting side-effects, especially in patients particularly concerned about self-appearance.

This study presents some limitations. First, the lack of a control group and the small size of the study cohort. Second, the diagnostic arthroscopy by clearing the joint from pro-inflammatory synovial fluid could be a confounding factor not allowing a clear understanding of the isolated efficacy of MFAT intra-articular infiltration. Nevertheless, authors believe arthroscopy is useful to better assess intra-articular environment and chondral lesions, to possibly address, within the same surgical session, associated lesions, and to reduce inflammatory milieu of the joint which could hamper treatment effect [33].

Within the study group we did not show correlation with age, BMI, previous knee surgery, ADSCs dose and cartilage damage degree status. It is unclear if this is the case due to a limited number of patients or to the actual absence of correlations. The positive finding of joint degenerative arrest on X-rays at the end of follow-up might be considered the effect of a disease modifying treatment, although the absence of MRI or arthroscopic evaluation to detect cartilage status could be considered a limitation. However, MRI is usually not sufficient for precise cartilage defect definition and a second-look arthroscopy was not performed for ethical reasons [51]. Finally, the lack of a follow-up evaluation in between 1 and 4 years did not allow to draw a precise conclusion on when the decline of outcome scores started. Notwithstanding, the mid-term follow-up represents a unique and major strength of the current study, documenting the relative effectiveness of the treatment up to 4 years.

Overall, considering the minimally invasive nature of the procedure, and the increasing number of active middle-aged knee OA patients with high functional needs who prefer to postpone TKR, the therapeutic benefits lasting up to 4 years seem promising. Further studies with higher patients’ number, a proper control group and a standardized treatment protocol are required to confirm these preliminary findings.

## Conclusions

MFAT intra-articular injection showed to be a safe therapeutic option with positive improvements up to 4 years of follow-up in 68% of patients with early to moderate knee OA. BMI, sex, age, ADSCs dose and previous surgeries did not correlate with the outcome, while synovitis may be predictive for treatment failure. These findings suggest MFAT could be a minimally invasive treatment with durable benefits at mid-term evaluation.

## Data Availability

The data sets used and/or analyzed during the current study are available from the corresponding author on reasonable request.

## Abbreviations

OA: Osteoarthritis
MFAT: Microfragmented Autologous Fat Tissue
TKR: Total knee replacement
PRP: Platelet-rich plasma
MSCs: Mesenchymal stem cells
ADSCs: Adipose tissue-derived stem cells
FDA: Food and Drugs Administration
EMA: European Medical Agency
RCTs: Randomized controlled trials
NSAIDs: Non-steroidal anti-inflammatory drugs
IRB: Institutional review board
STROBE: Strengthening the Reporting of Observational Studies in Epidemiology
K–L: Kellgren–Lawrence
MRI: Magnetic resonance imaging
ASA: American Society of Anaesthesiologists
ICRS: International Cartilage Repair Society
WOMAC: Western Ontario and McMaster Universities Arthritis Index Score
ROM: Range of motion
VAS: Visual Analogue Scale
MCII: Minimal Clinically Important Improvement
ANOVA: Analysis of variance
BMI: Body mass Index
